# Structured Diagnostic Interviews in Psychotherapy Training: Trainees’ Beliefs About Interviews and Their Relationship to Overall Interview Satisfaction

**DOI:** 10.32872/cpe.17321

**Published:** 2025-11-28

**Authors:** Sebastian Palmer, Bertram Walter, Christiane Hermann, Rudolf Stark, Andrea Hermann

**Affiliations:** 1 Department of Psychotherapy and Systems Neuroscience, Justus Liebig University Giessen, Giessen, Germany; 2 Cognitive-Behavioral Psychotherapy Outpatient Clinic, Justus Liebig University Giessen, Giessen, Germany; 3 Department of Clinical Psychology, Justus Liebig University Giessen, Giessen, Germany; Philipps-University of Marburg, Marburg, Germany

**Keywords:** structured diagnostic interviews, psychotherapy training, interview satisfaction, psychotherapeutic orientation, personality

## Abstract

**Background:**

Structured diagnostic interviews (SDIs) are frequently used in science and are highly recommended for diagnosing mental disorders in clinical practice. However, the actual SDI familiarity and use among psychotherapy practitioners is limited. To identify opportunities for training improvement and ensure a frequent SDI application by future practitioners, data on SDI experiences and beliefs among current psychotherapy trainees is essential.

**Method:**

*N* = 233 psychotherapy trainees completed an online survey that included questions about their SDI experiences, use, beliefs, and their estimation of patient SDI satisfaction and acceptance. In addition, adherence to psychotherapeutic orientation and personality factors were assessed. Correlation between SDI satisfaction and familiarity was computed. Multiple linear regression analysis was performed to predict trainees’ SDI satisfaction by beliefs about SDIs. Exploratory correlations between SDI satisfaction, adherence to psychotherapeutic orientations, and personality factors were analyzed.

**Results:**

SDI familiarity was significantly related to trainees’ overall SDI satisfaction. Both positive (e.g., “SDIs are efficient”) and negative (e.g., “SDIs disturb the relationship to patients”) beliefs about SDIs predicted trainees’ overall satisfaction. Small relationships were found between SDI satisfaction and adherence to psychotherapeutic orientation, but none to personality factors.

**Conclusion:**

Psychotherapy training programs should provide sufficient opportunity for SDI practice to promote trainee satisfaction. Training providers should address trainees’ beliefs and concerns, underline advantages of SDIs, and inform about actual SDI acceptance among patients to resolve prejudice. Trainees’ personality appears to be less relevant to SDI satisfaction, but further investigations are needed. The findings have important implications for overcoming barriers to the use of structured diagnostic interviews.

## Highlights

- Most psychotherapy trainees encounter SDIs, but experiences and use vary.
- Trainees’ level of SDI satisfaction is medium on average, but it positively relates to familiarity.
- Satisfaction is higher when SDIs are viewed as reliable and efficient.
- The views that SDIs are confusing and threaten the therapeutic relationship raise dissatisfaction.

Diagnostic decisions of mental health practitioners often deviate from the criteria of classification systems, increasing the risk for inaccurate diagnoses and suboptimal treatment recommendations (Rettew et al., 2009; Wolkenstein et al., 2011; Zimmerman & Mattia, 1999). Structured diagnostic interviews (SDIs) have been repeatedly proposed as a standard procedure to help diagnostic decision-making (Basco et al., 2000; Joiner et al., 2005; Miller et al., 2001). While broader SDIs, such as the Structured Clinical Interview for DSM-5 (SCID-5, First et al., 2016) assess a range of diagnoses, diagnosis-specific SDIs, like the Clinician-administered PTSD Scale for DSM-5 (CAPS, Weathers et al., 2013) focus on a single disorder. Previous evidence indicates that SDIs have high reliability, validity, and acceptance among patients (Margraf et al., 2017; Neuschwander et al., 2017; Osório et al., 2019; Suppiger et al., 2009).

Despite the advantages of SDIs, a substantial number of clinicians refrains from their regular use in clinical practice (Bruchmüller et al., 2011; Cook et al., 2017; Hatfield & Ogles, 2004; Jensen-Doss & Hawley, 2010). A survey by Bruchmüller et al. (2011) on the SDI practices of licensed psychotherapists revealed that, on average, SDIs are used in only 15% of patient encounters. The most common arguments against the use of SDIs include their lack of usefulness compared to clinicians’ own judgement, their length, and their potential harm to the therapeutic relationship, all of which predicted practitioners’ actual use of SDIs. In light of the acceptance and satisfaction rates among patients (Suppiger et al., 2009), Bruchmüller et al. (2011) concluded that practitioners tend to overestimate the negative and underestimate the positive impact of SDIs.

Interestingly, actual knowledge of SDIs among practitioners is limited. More than a third of the Bruchmüller et al. (2011) sample was barely or not at all familiar with SDIs, with SDI familiarity predicting SDI use. Similarly, Cook et al. (2017) found that practitioners who predominantly endorsed unstructured assessment practices were less likely to report training in standardized assessment, including standardized diagnostic interviews. These findings suggest that implementation of SDIs into early professional training is not only important for fostering competence, but also for ensuring their regular use. Psychotherapy training guidelines consider assessment competencies as crucial, yet they do not necessarily recommend specific diagnostic methods (Hatcher et al., 2013; Wright, 2022). Surveys among psychotherapy trainees and training providers indicate that a considerable number of trainees do not receive formal practical SDI training (Ingram et al., 2022; Mihura et al., 2017; Ponniah et al., 2011). Accordingly, there is a need for information about trainees’ actual experiences with and beliefs about SDIs that may help identifying facilitators and barriers to SDI training.

Another finding from the Bruchmüller et al. (2011) study is that a primary orientation in cognitive-behavioral therapy (CBT) positively predicted SDI use. Most likely, this is accounted for by CBT therapists’ more positive attitude towards symptom-based classification systems (Raskin et al., 2022). Additionally, the preferred theoretical orientations of psychotherapists have been found to be related to their personality. Poznanski and McLennan (2003) reported higher neuroticism in psychodynamic psychotherapists compared to CBT therapists, whereas the latter showed lower levels of openness. Ogunfowora and Drapeau (2008) found that conscientiousness positively predicted a CBT orientation in psychotherapy students and psychotherapists. To clarify whether trainees’ personality not only relates to broader theoretical orientation preferences, but also to evaluations of SDIs directly, a combined investigation is necessary. Results could inform the development of SDI training schedules that consider trainees’ individual differences, especially with respect to their personality. As personality has been shown to relate to professional development and well-being throughout training (Chapman et al., 2009; Evers et al., 2019), it might also be worth considering it when training and supervising student therapists in conducting an SDI.

Building upon previous findings on SDI practices and beliefs of practitioners (Bruchmüller et al., 2011; Cook et al., 2017), the present study investigated experiences with SDIs among German psychotherapy trainees. Participants completed an online survey on their SDI use, overall satisfaction, their beliefs about SDIs, their estimation of patients’ SDI satisfaction and acceptance, as well as personality factors and adherence to psychotherapeutic orientations. Extending the findings by Bruchmüller et al. (2011), the present study examined the relationships of psychotherapy trainees’ overall SDI satisfaction with specific positive and negative beliefs about SDIs and SDI familiarity. The following main hypotheses were tested:

1. There is a significant relationship between overall SDI satisfaction and SDI familiarity.
2. Overall SDI satisfaction is related to psychotherapy trainees’ agreement to positive and negative beliefs about SDIs.

In addition, exploratory analyses were performed to investigate the relationship between trainees’ SDI satisfaction, their adherence to psychotherapeutic orientations, and personality factors.

## Method

### Participants

**Figure 1:**
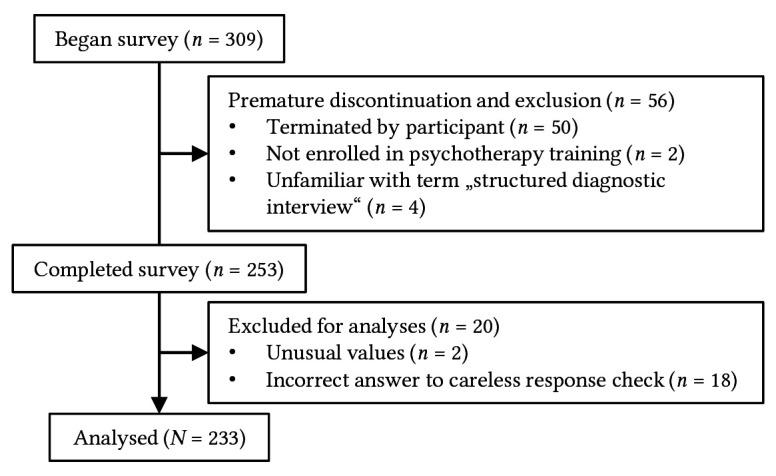
Participant Flow Chart

Participants were recruited among training institutions for post-graduate psychotherapy training in Germany (for further information see Procedure). Figure 1 shows participant flow.

The final sample for analysis consisted of *N* = 233 psychotherapy trainees. The mean age was *M* = 31.05 years (*SD* = 5.72). On average, participants were enrolled for *M* = 3.05 (*SD* = 2.02) years into their current training program and had *M* = 3.96 (*SD* = 3.39) years of experience in working with patients, with six participants reporting no prior patient experience. For further participant information see Table 1.

**Table 1:** Demographic and Training Information of Psychotherapy Trainees

Variable	*n*	%
Gender		
Female	202	86.7
Male	30	12.9
Diverse	1	0.4
Type of training		
Psychological psychotherapy	171	73.4
Child and adolescent psychotherapy	61	26.2
Other	1	0.4
Training orientation		
Behavioral therapy	208	89.3
Psychodynamic psychotherapy	10	4.3
Psychoanalytic psychotherapy	2	0.9
Systemic psychotherapy	9	3.9
More than one orientation	4	1.7
Study course prior to training		
Psychology	204	87.6
Medicine	1	0.4
Social pedagogy	11	4.7
Educational sciences	12	5.2
Other	10	4.3
Type of training institute		
University-based	119	51.1
Private	112	48.1
Other	2	0.9

*Note.* *N* = 233.

### Surveys and Measurements

At the beginning, the survey explained the term “structured diagnostic interview” and gave examples of common interview schedules (see Supplement 1). Subsequently, participants were asked about the context in which they learned or practiced SDIs, the total number of SDIs conducted, their familiarity with SDIs (1 = “not at all familiar” to 9 = “very familiar”), and their overall satisfaction with SDIs (0 = “not at all satisfied” to 100 = “totally satisfied”). The latter rating was adapted from the Interviewer Acceptance Questionnaire (Suppiger et al., 2009). Furthermore, participants rated their current frequency of use of different diagnostic information and methods to assess diagnoses during patient encounters (1 = “never” to 9 = “always”), and their estimated use of SDIs for diagnosing patients after completing training (1 = “never” to 9 = “always”).

The therapist version of the Patient Acceptance Questionnaire (PAQ, Bruchmüller et al., 2011; Suppiger et al., 2009) was included to measure participants’ estimation of patients’ SDI satisfaction and acceptance. First, participants estimated patients’ overall SDI satisfaction (0 = “not at all satisfied” to 100 = “totally satisfied”). Second, ten single items assessed participants’ estimate of patients’ acceptance. The single items include both positive (e.g., “In an SDI patients feel that the interviewer takes their problems seriously”) and negative statements (e.g., “Patients find SDIs exhausting”). Participants rated their endorsement of each item on a four-point scale (0 = “does not apply at all”, 1 = “somewhat applies”, 2 = “quite completely applies”, 3 = “completely applies”).

Additionally, participants rated their agreement to six positive and nine negative beliefs about SDIs (see Supplement 2). Eleven items (e.g., “SDIs help not to overlook anything”, “SDIs take too long”) were adapted from Bruchmüller et al. (2011). The first author formulated four additional items (e.g., “SDIs help to broaden one’s own diagnostic competence”, “Learning SDIs takes too much time”) based on feedback from participants of a psychotherapy training course. Participants rated their agreement to the presented beliefs on a nine-point scale (1 = “I totally disagree” to 9 = “I totally agree”). In addition, participants could provide further statements on SDIs.

Participants’ personality factors were measured using the German short version of the Big Five Inventory (BFI-K, Rammstedt & John, 2005). Based on the five-factor model of personality, the BFI-K contains the scales extraversion, agreeableness, conscientiousness, neuroticism, and openness. It consists of 21 items which participants rate on a five-point scale (1 = “strongly disagree” to 5 = “strongly agree”). The BFI-K has sufficient validity and reliability across different samples (Kovaleva et al., 2013; Rammstedt & John, 2005).

Finally, participants’ demographic information, including age, gender, previous study courses, and information on their current training program were assessed. Additionally, participants rated their adherence to different psychotherapeutic orientations (psychodynamic/ analytic, cognitive/ behavioral, and systemic psychotherapy) on a nine-point scale (1 = “not at all” to 9 = “very strongly”). These orientations were chosen because they are offered by state-approved post-graduate training institutes in Germany.

The survey included two instructed response items to check for careless responding. In the first item, participants were instructed to choose the option “slight agreement” on a five-point rating scale (“strong disagreement”, “slight disagreement”, “neutral”, “slight agreement”, “strong agreement”). The second item used a multiple-choice format with four options numbered 1 to 4, where participants were instructed to choose “Option 4”.

### Procedure

The data were collected between February and June 2023. The online survey was created using SoSci Survey (Leiner, 2024) and was made available on http://www.soscisurvey.de. The link to the survey was sent via e-mail to 172 state-approved training institutions for post-graduate psychotherapy training in Germany from all four major psychotherapeutic orientations (behavioral, psychodynamic, psychoanalytic, and systemic psychotherapy). Institutions were contacted either directly through the contact information on their website or through a mailing list. Contact persons were asked to disseminate the information and link to the survey among the institutions’ current psychotherapy trainees. Upon accessing the link, participants received participant information and actively consented to survey participation. Participants could enter a personal e-mail address for a chance to win one of five 20€ online vouchers after survey completion. E-mail addresses were saved separately from participants’ survey data to ensure anonymity.

### Statistical Analysis

All analyses were performed using IBM SPSS Statistics (Version 28). The main analyses were preregistered at *AsPredicted* (https://aspredicted.org/N3F_9T3). Secondary analyses as well as descriptive analyses regarding a further student sample, as described in the pre-registration, will not be reported in the present manuscript. Due to the composition of the sample and the results of the correlational analyses, the pre-registered test of regression and mediation models concerning personality factors and adherence to orientation were not computed. Only the results from exploratory correlational analyses are reported (see Results).

Significance level was set at α = .05 for all analyses. A two-tailed test of correlation between SDI familiarity and overall SDI satisfaction was computed. Additionally, a multiple linear regression model of overall SDI satisfaction on the agreement to 15 beliefs about SDIs was computed. All predictors were entered in a single step. A priori power analysis using G*Power (Faul et al., 2009) indicated that a minimum of *N* = 143 participants was needed for the regression analysis to detect a medium effect with power = .80. Small to moderate intercorrelations were observed between predictors, but mean VIF = 1.75 indicated no multicollinearity (see Supplement 3). Exploratory correlations between overall SDI satisfaction, the BFI-K subscales (Rammstedt & John, 2005), and the ratings of adherence to psychotherapeutic orientations were computed. Due to the negatively skewed distribution for the cognitive/behavioral and the positively skewed distribution of the psychoanalytic/psychodynamic orientation ratings, non-parametric Kendall’s Tau C correlations are reported.

The answers to the open question about further beliefs about SDIs were categorized by the first author. First, specific entries were categorized into positive and negative arguments. Second, specific topics were identified. The most frequent topics for both positive and negative arguments, along with examples of participant entries are reported.

## Results

### SDI Experience, Use, and Satisfaction

Participants commonly reported treatment of outpatients (70%) as a context for SDI encounters, followed by courses during their master’s program and training (67.4%), treatment of inpatients (50.6%), and research (28.3%). The estimated number of SDIs conducted by psychotherapy trainees ranged from zero to 200 interviews. Most trainees (42.1%) conducted 10 or fewer SDIs, with 13 participants reporting that they had never conducted an SDI before. The majority (66.5%) of participants stated that conducting SDIs was mandatory during their training program.

On average, trainees reported a medium level of overall SDI satisfaction (*M* = 60.43, *SD* = 20.83, range: 0 – 100) and endorsed a moderate level of SDI familiarity (*M* = 6.03, *SD* = 1.78, range: 1 – 9). A moderate positive correlation between overall SDI satisfaction and SDI familiarity was observed, *r*(231) = .41, *p* < .001.

**Figure 2:**
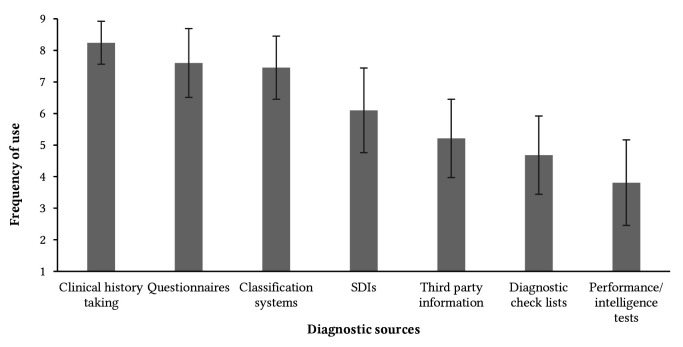
Psychotherapy Trainees’ Use of Diagnostic Sources for Diagnosing Patients *Note.* *n* = 227; SDI = structured diagnostic interview; frequency of use was rated from 1 (“never”) to 9 (“always”); error bars indicate standard deviations.

Concerning estimated SDI use after training (*M* = 5.79, *SD* = 1.81, range: 1 – 9), 2.6% of trainees stated that they would “never”, while 6.4% stated that they would “always” use an SDI for diagnosing patients. For diagnosing patients, trainees with patient experience (*n* = 227) reported to use clinical history taking most frequently, followed by questionnaires, classification systems, and SDIs (see Figure 2).

### Trainees’ Estimation of Patients’ SDI Satisfaction and Acceptance

Psychotherapy trainees estimated a medium level of overall SDI satisfaction among patients (*M* = 55.60, *SD* = 20.62, range: 0 – 95). The positive statements with the highest mean agreement ratings were the statement: “During an SDI, patients feel that the interviewer takes their problems seriously” (*M* = 1.82, *SD* = 0.86) and the statement: “After an SDI, patients think that the interviewer asked for enough detail to get an appropriate understanding of their situation” (*M* = 1.82, *SD* = 0.87). In contrast, psychotherapy trainees tended to only partially agree or disagree with the statement: “Patients have the feeling that they understand themselves and their problems better after an SDI” (*M* = 0.84, *SD* = 0.77). Among the negative items, the estimation that patients find SDIs exhausting received the highest mean agreement (*M* = 1.50, *SD* = 0.80). Descriptive statistics on all PAQ items are shown in Supplement 4.

### Prediction of Psychotherapy Trainees’ SDI Satisfaction by Beliefs About SDIs

The regression model of overall SDI satisfaction on beliefs about SDIs was significant, *F*(15, 217) = 17.01, *p* < .001. The whole model explained 54% of variance. Descriptive and regression statistics for all beliefs are shown in Table 2. Two of the positive beliefs (“SDIs are reliable sources of information”, “SDIs are efficient”) positively predicted overall SDI satisfaction, whereas two of the negative beliefs (“SDIs disturb the relationship to patients”, “SDIs are too confusing”) negatively predicted overall SDI satisfaction.

**Table 2:** Multiple Linear Regression of Psychotherapy Trainees’ Overall SDI Satisfaction on Beliefs About SDIs

Predictor	*M* (*SD*)	*B* (*SE*)	95% CI	β	*T*
Positive beliefs					
SDIs help to not overlook anything.	7.33 (1.51)	0.80 (0.79)	[-0.76, 2.36]	0.06	1.02
SDIs help to broaden one’s own diagnostic competence.	6.67 (1.88)	0.21 (0.63)	[-1.04, 1.45]	0.02	0.33
SDIs are reliable sources of information.	6.49 (1.47)	3.01 (0.97)	[1.09, 4.93]	0.21	3.1**
SDIs secure the quality of treatment.	6.12 (1.99)	0.21 (0.69)	[-1.14, 1.56]	0.02	0.31
SDIs help with treatment planning.	5.67 (2.07)	-0.33 (0.59)	[-1.49, 0.83]	-0.03	-0.56
SDIs are efficient.	5.19 (1.72)	2.60 (0.73)	[1.15, 4.04]	0.21	3.54***
Negative beliefs					
SDIs take too long.	6.73 (1.86)	-1.34 (0.71)	[-2.75, 0.06]	-0.12	-1.89
My clinical judgment is more useful to me than an SDI.	4.61 (2.12)	-0.87 (0.62)	[-2.10, 0.35]	-0.09	-1.40
SDIs are too confusing.	4.58 (2.10)	-1.74 (0.61)	[-2.94, -0.55]	-0.18	-2.87**
SDIs are unpleasant for patients.	4.50 (1.95)	1.08 (0.67)	[-0.23, 2.4]	0.10	1.62
SDIs are too detailed.	4.40 (1.95)	1.16 (0.65)	[-0.13, 2.44]	0.11	1.78
Learning SDIs requires too much time.	4.11 (2.13)	-0.94 (0.50)	[-1.93, 0.06]	-0.10	-1.86
SDIs disturb the relationship to patients.	3.73 (2.10)	-2.36 (0.66)	[-3.67, -1.06]	-0.24	-3.57***
SDI questions are barely understandable.	3.36 (1.67)	-0.47 (0.70)	[-1.84, 0.90]	-0.04	-0.68
Information from SDIs are not relevant for treatment.	2.27 (1.58)	-0.58 (0.77)	[-2.09, 0.93]	-0.04	-0.76

*Note. N* = 233; *R*^2^ = .540; *SE* = standard error; CI = confidence interval; SDI = structured diagnostic interview; trainees’ beliefs about structured diagnostic interviews were rated on a scale from 1 (“I totally disagree”) to 9 (“I totally agree”).

**p < .01. ***p < .001.

### Trainees’ Answers to Open Question

Participants entered 31 further arguments for and against SDIs. Among the positive arguments, the identified topics were: benefit for patients and the therapeutic relationship (e.g., “the diagnosis [using an SDI] induces confidence that the therapist relies on scientific evidence, and not arbitrary; the patient can rely on the result”), and benefit for determining accurate diagnoses (e.g., “in particular cases [the SDI] uncovered symptoms in the past”). Among the negative arguments, the most common topics included: weakness of the interview itself (e.g., “structured interviews are too undynamic”), lack of benefit for determining a diagnosis (e.g., “[…] the interview only showed that there is psychological distress, it was not helpful diagnostically”), detrimental effect on patients and the therapeutic relationship (e.g., “SDIs can irritate patients ([they create the] impression that one’s very sick if one has to answer such different questions)”), high effort (e.g., “SDI require a lot of time/resources, that, to my experience, are not available in the daily clinic routine”), and lack of appropriate compensation (e.g., “structured interviews are not sufficiently rewarded during training (neither monetary, nor for the progress of training), which can be frustrating”).

### Relationship Between Personality Factors, Adherence to Psychotherapeutic Orientations, and SDI Satisfaction

The exploratory correlational analysis included only participants that reported experience in working with patients (*n* = 227). A small negative relationship was found between psychotherapy trainees’ overall SDI satisfaction and the adherence to a psychoanalytic/psychodynamic orientation, τ*_c_* = -.14, *p* = .005. In contrast, a small positive relationship was found between psychotherapy trainees’ overall SDI satisfaction and the adherence to a cognitive/behavioral orientation, τ*_c_* = .11, *p* = .005. No relationships were found between overall SDI satisfaction and any personality factor. Full results of the correlational analyses are reported in Supplement 5.

## Discussion

The results of the present study indicate that current psychotherapy trainees have encountered SDIs in different contexts throughout training, predominantly during outpatient treatment. Compared to the practitioner sample from Bruchmüller et al. (2011), the degree of familiarity with SDIs was higher, which might reflect a growing importance of SDIs in clinical practice and corresponding changes in the curricula of psychotherapy training. Still, one third of the trainees reported that they were not required to use SDIs during training, and the number of conducted SDIs varied considerably. In line with findings from US samples, SDIs are less frequently used than self-report questionnaires, possibly due to the availability and feasibility of the latter, even though they lack usefulness for diagnostic classification (Ingram et al., 2022; Mihura et al., 2017). The relationship between SDI satisfaction and SDI familiarity underlines the importance of sufficient SDI coverage during training. However, further longitudinal investigations need to clarify the direction of the relationship: while a higher SDI familiarity could lead to higher satisfaction, it is also plausible that a satisfying initial SDI experience could motivate trainees to become more familiar with SDIs.

The results on the beliefs about SDIs show that trainees recognize the advantages of SDIs for practice and professional development, but negative beliefs persist to some extent. Moreover, regression analysis revealed that beliefs differed in their impact on trainees’ overall SDI satisfaction. The positive association between SDI satisfaction and the beliefs that SDIs are reliable and efficient implies that educating trainees about the existing evidence on the reliability and validity of SDIs, ideally in the early phases of training, could strengthen their trust in the method and their confidence in their clinical decisions (Margraf et al., 2017; Osório et al., 2019). In contrast, SDI satisfaction was negatively related to the belief that SDIs harm the therapeutic relationship. Additionally, trainees estimated patients’ overall SDI satisfaction as moderate, on average, which is similar to the practitioner estimation reported by Bruchmüller et al. (2011). Furthermore, 45.9% of the trainees quite or completely agreed that patients find SDIs exhausting. Given these results, it is crucial that trainees learn about evidence of patients’ SDI satisfaction and acceptance. Particularly, the finding that most patients view the relationship to interviewers as positive might challenge the misconception that SDIs inherently interfere with the therapeutic relationship (Neuschwander et al., 2017; Suppiger et al., 2009). Additionally, individual patients’ SDI acceptance and satisfaction ratings might help trainees and supervisors to identify difficulties and opportunities for improvements in future SDI applications.

Furthermore, a negative relationship was observed between SDI satisfaction and the belief that SDIs are too confusing. The confusion and related dissatisfaction may partly arise from reservations about standard classification systems. Even though classification systems are widely used and considered helpful, many practitioners would prefer concise manuals with fewer diagnostic categories (Evans et al., 2013; Reed et al., 2011). Moreover, the definition and validity of diagnostic criteria of certain disorders, e.g. generalized anxiety disorder, have been repeatedly challenged and debated (Andrews et al., 2010; Ruscio et al., 2024). Confusion could be reduced if trainees receive ongoing support during the study of the diagnostic criteria and the structure of SDIs. Moreover, trainees should have the opportunity to start practicing with non-clinical interviewees. The combination of theoretical and role play sessions in an SDI course for clinical psychology master’s students has already proven to be highly accepted by participants (Bonnin et al., 2024). Subsequently, a guided and supervised approach could ensure that trainees gradually move from clearly circumscribed to more complex cases (e.g., with higher comorbidity). Notably, institutions should also consider how trainees could be remunerated more adequately (e.g., additional therapy sessions and credit for training, provide SDI-specific supervision), thus encouraging regular SDI use.

Lastly, the belief that SDIs take too long did not significantly predict SDI satisfaction but had a high mean agreement in the present sample. Similarly, additional participant statements criticized SDIs as being too effortful for clinical practice. The perceived impracticability of SDIs is a common argument against their use, yet many practitioners are barely familiar with SDIs (Bruchmüller et al., 2011; Cook et al., 2017). As interview duration likely decreases along with SDI experience and familiarity, the view that SDIs take too long might change accordingly. Building on the present finding that SDI familiarity positively relates to overall satisfaction, future studies might prospectively assess the duration of individual SDIs and trainees’ estimated SDI practicability over the course of training.

In addition to the impact of trainees’ beliefs, the exploratory correlational analyses revealed relationships between SDI satisfaction and adherence to cognitive/behavioral as well as psychodynamic/psychoanalytic orientations. The positive relationship between satisfaction and adherence to a cognitive/behavioral orientation is in line with the results reported by Bruchmüller et al. (2011). As noted earlier, the relationship is likely accounted for by CBT therapists’ more positive attitude towards the underlying classification systems (Raskin et al., 2022). For a direct test of this assumption, future studies should jointly assess therapists’ attitudes towards classification systems and beliefs about SDIs. In contrast to orientation adherence, trainees’ personality appeared to be of less importance for their SDI evaluation, as no significant relationships were found. While personality factors relate to orientation preferences and the underlying general approach to psychotherapy, endorsement and evaluation of SDIs could be more closely related to specific behaviors and skills that are relevant in the therapeutic process. For instance, since the application of an SDI is a form of therapist-patient-interaction, interpersonal behaviors of psychotherapy might be worth investigating in this context. It is important to note that comparisons to previous studies are limited by the high proportion of CBT trainees in the present sample (Bruchmüller et al., 2011; Poznanski & McLennan, 2003), and the small effects from exploratory analyses need to be interpreted with caution.

### Study Limitations

The present study has limitations that need to be considered. First, the cross-sectional design does not allow for conclusions regarding possible long-term effects of SDI training (e.g., on actual SDI use after training). Second, certain survey items might have been insufficient. Even though most SDI beliefs were endorsed previously (Bruchmüller et al., 2011), the additional arguments reported by participants suggest that there are more issues relevant to SDI satisfaction. Single items of adherence to psychotherapeutic orientations were used as ecological and integrative measures but may have failed to capture all aspects relevant to orientation adherence. Trainees that are at an early stage of training might have had more difficulties providing accurate answers due to little experience. Third, correlational and exploratory analyses somewhat limit the conclusions that can be drawn. However, the present study has important implications for clinical practice and future research despite this methodological limitation, as it is the first to provide evidence on SDI beliefs and satisfaction among trainees. Finally, as most participants were psychologists in CBT training, the present results may not generalize to trainees with other training orientations and professional backgrounds. Yet, importantly, the present sample is still representative of the current trainee population in Germany, as most psychologists choose a post-graduate training in CBT (Rabe-Menssen et al., 2021).

### Summary and Perspective

The present study is the first to provide evidence for the impact of beliefs about SDIs on psychotherapy trainees’ overall SDI satisfaction. The results imply that training programs need to increase their efforts to implement SDI training and provide opportunities for SDI application in different settings to strengthen trainees’ SDI familiarity. While considering trainees’ needs and concerns (e.g., time constraints, limited compensation), programs need to inform trainees about the evidence on SDIs that highlight diagnostic advantages and common misconceptions, especially regarding their impact on the therapeutic relationship (Neuschwander et al., 2017; Suppiger et al., 2009). Longitudinal studies are needed to determine the impact of SDI training experiences on practitioners’ SDI use following training. In addition to overall SDI satisfaction, it would be informative to assess trainees’ immediate response to individual SDIs, thus allowing for direct comparisons to previous studies on interviewer satisfaction (Neuschwander et al., 2017; Suppiger et al., 2009). This approach would also enable tracking changes in SDI satisfaction and help determine the number of conducted SDIs required for sufficient experience to benefit from SDIs’ advantages. When investigating relations of SDI endorsement and evaluations with individual characteristics of trainees, research might focus more on specific behaviors and skills, as they are possibly more relevant than personality factors, and can be directly addressed and monitored during training. Finally, future evaluations of SDI satisfaction should include trainees with different professional backgrounds and orientations to account for a wider trainee population.

## Supplementary Materials

The Supplementary Materials for the present manuscript include:

- Preregistration (Palmer et al., 2022S)
- Additional Information (Palmer et al., 2025S):
  - *Supplement 1*: Survey Introduction
  - *Supplement 2*: Beliefs About Structured Diagnostic Interviews
  - *Supplement 3*: Predictor Intercorrelations and Multicollinearity Statistics for Multiple Linear Regression of Overall SDI Satisfaction on Beliefs About SDIs
  - *Supplement 4*: Descriptive Statistics for the Patient Acceptance Questionnaire – Therapist Version
  - *Supplement 5*: Correlations Between Psychotherapy Trainees’ Overall SDI Satisfaction, Personality, and Adherence to Theoretical Orientations

PalmerS.
WalterB.
HermannC.
StarkR.
HermannA.
 (2022S). Acceptance and experience with structured interviews
[Preregistration; AsPredicted ID: #95,997]. PsychOpen. https://aspredicted.org/N3F_9T3


PalmerS.
WalterB.
HermannC.
StarkR.
HermannA.
 (2025S). Supplementary materials to "Structured diagnostic interviews in psychotherapy training: Trainees’ beliefs about interviews and their relationship to overall interview satisfaction"
[Additional information]. PsychOpen. 10.23668/psycharchives.21371
PMC1292318341725695

## Data Availability

The data and materials of this study are available on request from the corresponding author.

## Reporting Guidelines

The present manuscript was written in accordance with the JARS-Quant reporting standards for non-experimental designs.

## Preregistration

The main analyses of the present study were preregistered at *AsPredicted:* https://aspredicted.org/N3F_9T3

## References

[sp1_r1] Palmer S. Walter B. Hermann C. Stark R. Hermann A. (2022 S). Acceptance and experience with structured interviews [Preregistration; AsPredicted ID: #95,997]. PsychOpen. https://aspredicted.org/N3F_9T3

[sp1_r2] Palmer S. Walter B. Hermann C. Stark R. Hermann A. (2025 S). Supplementary materials to "Structured diagnostic interviews in psychotherapy training: Trainees’ beliefs about interviews and their relationship to overall interview satisfaction" [Additional information]. PsychOpen. 10.23668/psycharchives.21371 PMC12923183 41725695

